# Comparison of the Victorian emergency minimum dataset (VEMD) human intent coding descriptor to medical records in discriminating suicide attempts for emergency presentations to Eastern Health Psychiatric Triage, Victoria, Australia

**DOI:** 10.1177/18333583251360616

**Published:** 2025-09-04

**Authors:** Tianqi Hang, Stanley Innes, Judith Hope

**Affiliations:** 1 Adult Mental Health and Wellbeing Program, Eastern Health, Australia; 2 Eastern Health Clinical School, Monash University, Australia; 3 Centre for Mental Health Education and Research, Delmont Private Hospital, Australia

**Keywords:** suicide attempted, emergency medical services, medical records, data accuracy, mental health services, health information management

## Abstract

**Background:**

Administrative health data are widely used for suicide attempt surveillance yet concerns remain about accuracy. The Victorian Emergency Minimum Dataset (VEMD) Human Intent Descriptor is an administrative coding system for classifying self-harm and suicidality in emergency department (ED) presentations. This study evaluates its accuracy in detecting suicide attempts by comparing it to clinician-applied Columbia Classification Algorithm of Suicide Assessment (C-CASA) ratings from medical records.

**Method:**

This cross-sectional validation study examined 607 ED presentations referred to psychiatric triage across three hospitals in August 2020. C-CASA classifications were compared with corresponding VEMD Human Intent Descriptor data. Sensitivity, specificity, predictive values, likelihood ratios, and Cohen’s kappa were calculated. Receiver operating characteristic (ROC) curves assessed overall discrimination.

**Results:**

The VEMD descriptor demonstrated high specificity (99.0%) but low sensitivity (25.0%–27.3%), indicating many false negatives. The ROC analysis showed poor discriminatory ability (area under the curve = 0.62–0.63). Forty percent of missed cases were captured in ED diagnoses, highlighting gaps in coding accuracy.

**Conclusion:**

While the VEMD descriptor reliably confirms suicide attempts, its poor sensitivity limits its utility for surveillance. Findings underscore the need for improved coding protocols and alternative detection strategies to enhance suicide attempt surveillance in ED settings.

## Introduction

Suicide prevention has become a significant area of public health policy and funding, with the Commonwealth Department of Health developing a National Suicide Prevention Program dedicated to suicide prevention frameworks and action (Australian Government National Mental Health Commission, 2014). This is understandable given that suicide continues to be a significant cause of death in Australia (Australian Bureau Statistics, 2023). The national age-standardised rates have remained unchanged over the past 10 years at a rate of 12 deaths per 100,000 population.

Emergency departments (EDs) are a key site of care for those experiencing suicidal ideation or behaviour. It is now well known that persons who present to ED with suicidal ideation or attempts are at high risk of death by suicide (Goldman-Mellor et al., 2019). This is particularly true in the first month after presentation for those who are admitted to psychiatric facilities (Chung et al., 2017). An accurate understanding of the rates of ED presentations are important for suicidal behaviour surveillance and evaluating the effectiveness of suicide prevention interventions. Concerns have been raised over electronic health records data quality to improve their efficiency, transparency, comparability and interoperability (Lewis et al., 2023).

In recent decades, research on suicidal behaviour has increasingly used administrative databases to provide information on suicide attempts and deaths (Nevarez Flores et al., 2024). Studies have highlighted the limitations of using administrative coding systems, such as the International Statistical Classification of Diseases and Health Related Problems (ICD), to detect suicide attempts in ED settings. Research has consistently shown that ICD clinical codes have poor sensitivity for identifying suicidal behaviour (Bethell & Rhodes, 2009; Rhodes et al., 2002; Stanley et al., 2018; Walkup et al., 2012). For example, studies in Canada and Australia comparing ICD clinically coded data to detailed medical records found that over half of actual suicide attempts were not captured by ICD clinical coding, with some studies reporting sensitivities as low as 18% (Randall et al., 2017; Sveticic et al., 2020). These findings underscore the challenges of relying on administrative data for suicide surveillance and highlight the importance of validation studies comparing such coding systems to clinical documentation.

One administrative database in Victoria, Australia, is the Victorian Emergency Minimum Dataset (VEMD) and comprises administrative and clinical data for all presentations at Victorian public EDs (Victorian Department of Health and Human Services, 2017–2020). It is used for health service planning, policy assessment, clinical research, quality improvement and patient management. The VEMD Injury Surveillance dataset items are mandatory items, collected at triage when an injury has occurred and contain within it, the human intent specification. This records the clinician’s assessment of the most likely human intent in the occurrence of the injury or poisoning (Department of Health and Human Services, Victoria, Australia, 2020–2021). The research usefulness of an administrative database relies on the completeness and accuracy of the data. Factors have been identified that impact on VEMD data quality including time constraints, data entry training and formal orientation (Marson et al., 2005). Another study explored VEMD data quality by comparing it to medical records for acute cardiovascular conditions and unspecific chest pain and found varying concordance rates between VEMD items suggesting further validation was warranted (Bray et al., 2020). No previous studies have involved the Human Intent descriptor.

To evaluate the accuracy of administrative coding systems in detecting suicide attempts, a reliable reference standard is required. The Columbia Classification Algorithm of Suicide Assessment (C-CASA) is a clinician-applied tool developed to systematically classify suicidal and self-harm behaviours based on clinical documentation. It distinguishes between suicidal behaviour, non-suicidal self-injury and indeterminate intent (Posner et al., 2007). C-CASA has been used as a gold standard in previous validation studies of administrative data coding (Randall et al., 2017). In this study, C-CASA classifications were applied retrospectively to medical records by a trained clinician to serve as a clinical benchmark. Its terminology is endorsed by expert opinion (Meyer et al., 2010).

This study aimed to evaluate the accuracy of administrative coding data in identifying suicide attempts among ED presentations by

1. Comparing administrative and clinical data: Examine the agreement between the VEMD Human Intent Descriptor (administrative coding) and C-CASA classification (clinical documentation) in identifying suicide attempts among ED presentations.
2. Evaluating the performance of the VEMD Human Intent Descriptor: Assess the ability of the VEMD Human Intent Descriptor to accurately detect suicide attempts by evaluating its sensitivity, specificity, predictive values, likelihood ratios and overall accuracy compared to clinician-rated C-CASA classifications.

The intent was to inform future service improvement, research and policy-making.

## Method

This cross-sectional validation study was conducted to evaluate the data quality properties of the VEMD suicide data in adults with various level of suicidality identified from their clinical medical records from the ED setting. Ethics approval was obtained from Eastern Health HREC (Reference LR21-008).

### Participants

The study population data were drawn from one month (June 2020) of all clinical presentations to the Psychiatric Triage of three Eastern Health hospital Emergency Departments, which covered a catchment area of over 800,000 persons.

### Sample size

One week of Eastern Health ED data were analysed to determine the number and type (attempt vs ideation) of suicidal presentations to inform a sample size calculation for the study. The rate of suicide attempt in persons referred to Psychiatric Triage was 19% as per C-CASA; VEMD indicated no more than 11%. We assumed a Cohen’s *d* of 0.08, a power of 0.8 and an alpha level of 0.05 and a small correlation size; thus, the sample size needed was a minimum of 270 cases (Lehr, 2001).

### Measures

*The Human Intent Descriptor* is part of the VEMD Injury Surveillance dataset mandatory items that are collected at triage when an injury has occurred and completed before discharge from the ED. Within it, the human intent specification records the ED clinician’s assessment of the most likely human intent in the occurrence of the injury or poisoning. The code descriptors categorise self-harm into suicide attempt, non-suicidal self-injury or intentional self-harm with indeterminable suicidal intent

#### Columbia classification algorithm of suicide assessment (C-CASA)

The C-CASA has demonstrated excellent inter-rater reliability for use when coding for suicide and self-harm (Posner et al., 2007; Randall et al., 2017). The C-CASA defines a *suicide attempt* as a “potentially self-injurious behaviour associated with at least some intent to die, as a result of the act” (Posner et al., 2007:1037). This is distinguished from the *preparatory act* category, where the individual “takes steps to injure him/herself” (with at least some intent to die) but is “stopped by self or others from starting the self-injurious act, before the potential for harm has begun” (Posner et al., 2007: 1037).

**Figure 1.**
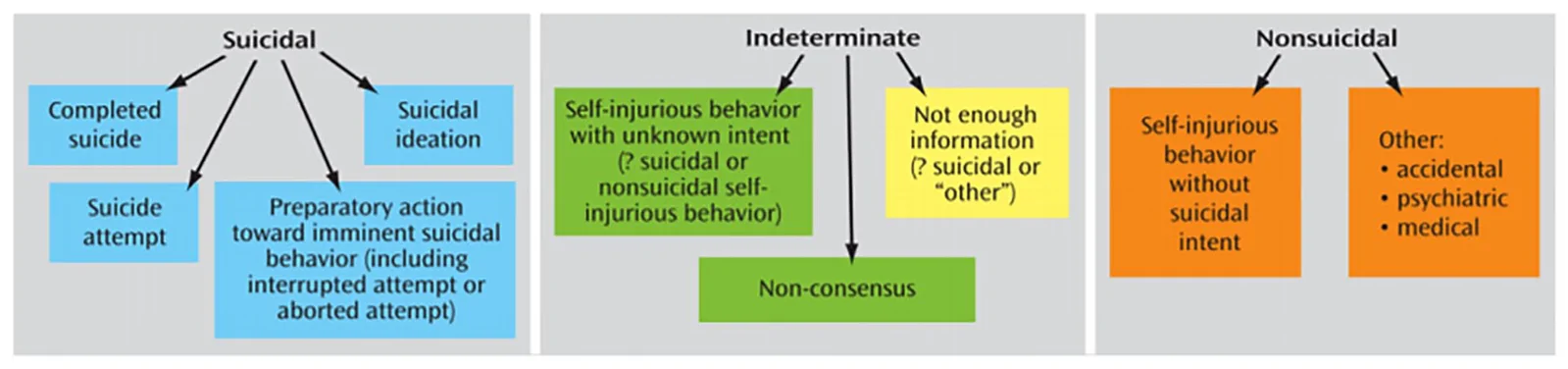
C-CASA suicidality classification scheme, extracted from Posner et al. (2007).

For the purpose of this study, two definitions of suicide attempt were applied for the purpose of predictive statistics; the *broad definition* included both C-CASA “suicide attempt” and “preparatory act” categories, whereas the *narrow definition* included C-CASA “suicide attempt” only (see Appendix 1). Suicidal ideation with no preparatory act or imminence was excluded from both definitions (Figure 1).

### Procedure

The consumer sample was derived from 1 month of ED contacts from the Victorian statewide mental health electronic database (Client Management Interface (CMI)) for June 2020. Patient identifiers (Name and UR Numbers) were extracted for their corresponding VEMD data. A research clinician (lead author) then sourced the complete matching electronic medical records from all treating medical and allied health practitioners and interrogated them to rate each case using the Columbia Classification Algorithm of Suicide Assessment (C-CASA) while being blinded to the VEMD data.

The human intent descriptor in the VEMD, if present, was then compared against the C-CASA coding for each presentation, for both the broad and narrow definitions for suicide attempt. For each presentation deemed a false negative, the author further checked if the ED clinician’s diagnosis captured the suicide attempt where the human intent descriptor did not.

### Data analysis

The agreement of the VEMD human intent descriptor from C-CASA classification (for broad and narrow definitions, respectively) was assessed using the Cohen’s kappa statistic. Predictive statistics and their 95% confidence intervals were derived, including sensitivity, specificity, positive predictive value (PPV) and negative predictive value (NPV), likelihood ratios and Cohen’s kappa. Receiver operating characteristic (ROC) curves were generated to assess overall discrimination.

## Results

All mental health CMI consumer ED contacts were obtained for the three Eastern Health EDs for August 2020 and any duplicate records removed. The study sample consisted of 607 unique presentations from the three hospitals.

### Administrative data compared to clinical data

Both the broad and narrow definitions of suicide attempt were used to compare the VEMD human intent descriptor against C-CASA classifications (Table 1). Among the 607 total ED presentations, 30 cases (4.9%) were correctly identified as suicide attempts by both the VEMD and C-CASA (true positives), while 482 cases (79.4%) were correctly identified as non-suicide attempts by both measures (true negatives). However, 90 cases (14.8%) were classified as suicide attempts by C-CASA but were not identified by the VEMD descriptor (false negatives). This suggests a substantial proportion of suicide attempts were missed by the VEMD coding. Importantly, 36 of these false-negative cases (40%) had an ED diagnosis that captured suicidality, typically recorded as “Suicide attempt without injury” or “Suicidal ideation.” Conversely, five cases (0.8%) were coded as suicide attempts in the VEMD but were not classified as such by C-CASA (false positives), indicating potential overclassification in a small subset of cases.

**Table 1.** Results using the broad definition compared to narrow definition.

	Broad definition *n* (%)	Narrow definition n (%)
Total cases	607	607
True negative	482 (79.4)	492 (81.1)
True positive	30 (4.9)	30 (4.9)
False negative	90 (14.8)	80 (13.2)
False positive	5 (0.8)	5 (0.8)
False negative – does ED Diagnosis capture suicide attempt?		
Yes	36	30
No	54	50

When using the narrow definition of suicide attempt (which excludes preparatory acts), 10 cases were reclassified as non-suicide attempts, reducing the false-negative count to 80 (13.2%). However, the number of true positives and false positives remained unchanged and suggest that the VEMD human intent descriptor did not consistently capture cases categorised under the C-CASA preparatory act category.

### Performance of the VEMD human intent descriptor

Table 2 summarises the performance of the VEMD human intent descriptor in detecting suicide attempts compared to the C-CASA classification, using both broad and narrow definitions. The results highlight that while the VEMD descriptor has high specificity (99.0%), meaning it is highly accurate in identifying cases that are not suicide attempts, its sensitivity remains low (25.0% for the broad definition and 27.3% for the narrow definition). This indicates that using the VEMD coding alone will result in a considerable number of suicide attempts going undetected.

**Table 2.** Statistics calculations for the broad and narrow definitions of suicide attempt.

	Broad definition % (95% CI)	Narrow Definition % (95% CI)
Sensitivity	25.0 (17.6% to 33.7%)	27.23 (19.2% to 36.6%)
Specificity	99.0 (97.6% to 99.7%)	99.0 (97.7% to 99.7%)
Positive likelihood ratio	24.35 (9.65 to 61.44)	27.11 (10.76 to 68.30)
Negative likelihood ratio	0.76 (0.68 to 0.84)	0.73 (0.65 to 0.82)
Condition prevalence	19.8 (16.7% to 23.2%)	18.1 (15.1% to 21.4%)
PPV	85.7 (70.4% to 93.8%)	85.7 (70.4% to 93.8%)
Negative predictive value (NPV)	84.2 (82.8% to 85.6%)	86.0 (84.6% to 87.3%)
Accuracy	84.4 (81.2% to 87.2%)	86.0 (83.0% to 88.7%)
Cohen’s Kappa	0.33 (0.23 to 0.42)	0.36 (0.26 to 0.46)

PPV: positive predictive value; NPV: negative predictive value.

The positive predictive value (PPV) of 85.7% suggests that when a case is identified as a suicide attempt by the VEMD, it is likely to be correct. However, the negative predictive value (NPV) of 84.2% to 86.0% reflects that many cases classified as non-attempts may still include undetected suicide attempts. Overall, the agreement between the two measures, as indicated by Cohen’s kappa (0.33–0.36), falls within the “fair” range, reinforcing concerns about the reliability of VEMD data as a screening tool for suicide attempts.

A sensitivity analysis excluding ambiguous cases showed no significant change in sensitivity but led to a small increase in specificity and PPV, reinforcing the finding that the VEMD descriptor is better suited for confirming suicide attempts rather than identifying them comprehensively. These findings underscore the limitations of relying on administrative coding alone for suicide attempt surveillance and highlight the need for improved data collection processes or alternative detection methods.

**Figure 2.**
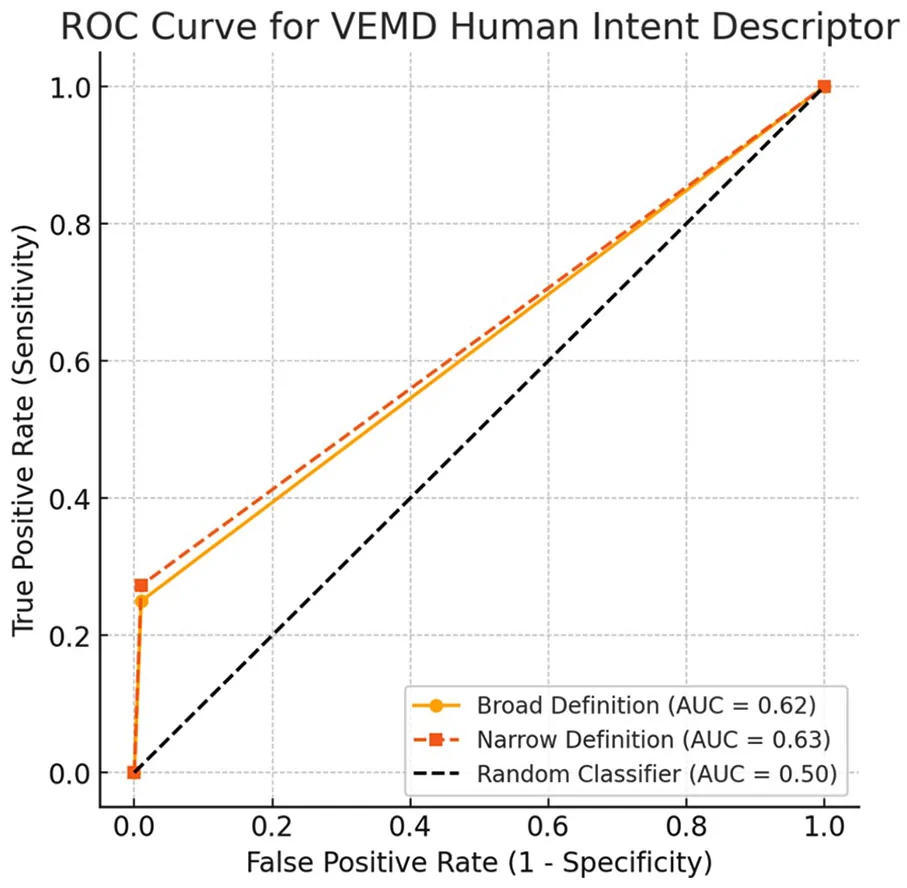
ROC curve for VEMD human intent descriptor. ROC: receiver operating characteristic; VEMD: Victorian emergency minimum dataset.

To further visualise the properties of the VEMD, a ROC was calculated. The area under the curve (AUC) and values are displayed in Figure 2. These AUC values indicate that the VEMD descriptor has lower levels of discriminatory ability in detecting suicide attempts, as an AUC closer to 1.0 represents a strong classifier, while 0.50 represents a random classifier. Further, a sensitivity analysis was conducted but excluding all 27 ambiguous cases. No difference in sensitivity scores were found. However, specificity scores increased to 99.8% (*both definitions*), PPV increased to 96.8% (both definitions) and NPV decreased to 83.6% (*broad definition*) and 85.4% (*narrow definition*).

## Discussion

This study evaluated the accuracy of the VEMD human intent descriptor in detecting suicide attempts in ED presentations by comparing it against the C-CASA classification system applied to medical records. The results demonstrated that while the VEMD descriptor has high specificity, indicating it reliably identifies cases that are not suicide attempts, its low sensitivity means that a significant proportion of actual suicide attempts go undetected. The ROC analysis further highlights its low levels of discriminatory ability, reinforcing concerns about the reliability of administrative coding for suicide attempt surveillance.

### Comparison of administrative and clinical data

A key finding was the poor agreement between the VEMD human intent descriptor and C-CASA classifications, with 14.8% of cases identified as suicide attempts in medical records not captured by the VEMD descriptor. Importantly, 40% of these false-negative cases were recorded in ED diagnosis fields as “Suicide attempt without injury” or “Suicidal ideation,” suggesting that suicidality or a suicide attempt was recognised clinically but not consistently coded in the administrative dataset. Known factors that contribute to inconsistent coding are staff inaccurately charting the occurrence of suicidal behaviour as a result of human error and/or staff inexperience with VEMD data (Randall et al., 2017). ED staff (medical, nursing, clerical) may also struggle with time constraints, software problems and lack of formal orientation and training in VEMD data entry (Marson et al., 2005). Additionally, these staff are sometimes not mental health trained and may be impacted by gaps in administrative data such as limited information and communication on ED triage presenting complaint (Phillips et al., 2015; Sveticic et al., 2020). This possibility may explain in our study why some cases classified as “preparatory acts” in C-CASA were not recorded in VEMD. A longitudinal study exploring Eastern Health ED staff views, attitudes and behaviours when coding VEMD data entry would allow insight into which of these reasons contributed to the observed detection rates.

### Performance of the VEMD human intent descriptor

Although the VEMD descriptor reliably identified non-suicide attempts, its low sensitivity suggests it is not suitable as a screening tool. The high specificity and positive predictive value indicate that when the VEMD classifies a case as a suicide attempt, it is likely correct. However, the negative predictive value suggests that many cases classified as non-suicide attempts in VEMD may still include undetected suicide attempts. The ROC analysis further demonstrated poor discriminatory ability, with an AUC of 0.62–0.63, reinforcing that the VEMD descriptor is more effective for confirming suicide attempts rather than detecting them comprehensively. Sensitivity analyses excluding ambiguous cases led to minor increases in specificity and PPV, but did not improve sensitivity, underscoring the structural limitations of VEMD coding.

The underreporting of suicide attempts in administrative data has important implications for suicide prevention efforts. If suicide attempt rates are systematically underestimated, health services may fail to allocate adequate resources for crisis response and suicide prevention interventions. Furthermore, the use of administrative data for evaluating suicide prevention initiatives could be compromised by incomplete case identification. Despite the low sensitivity of the VEMD descriptor, Cohen’s kappa values (0.33–0.36) suggested only “fair” agreement between VEMD and C-CASA classifications. This reflected moderate consistency between the two measures but also highlights significant discrepancies in how suicide attempts are recorded in administrative versus clinical data. Importantly, narrowing the definition of suicide attempt (by excluding preparatory acts) had only a minor effect on kappa values, reinforcing that classification issues extend beyond definitional differences and are likely linked to broader limitations in coding practices.

The method in our study of comparing administrative data (VEMD Human Intent Descriptors) to clinical records resulted in higher levels of sensitivity when compared to another Australian study that utilised ICD-10-AM clinical codes (Sveticic et al., 2020). While similar definitions of suicide attempt were used in both studies, Sveticic et al (2020) excluded cases categorised as ambiguous intent. We performed both analysis (including and excluding ambiguous cases) and found that it only altered sensitivity levels and other predictive statistics by a small margin. Also both studies found very similar suicide attempt and suicidal ideation rates (Sveticic et al., 2020). These findings demonstrate the magnitude of suicidal ideation and behaviour in the ED within a local region and may help inform whether current demand exceeds the capacity of health and community services to respond effectively. This taken with the moderate level of agreement observed underscores the need for enhanced standardisation in ED coding practices to reduce variability and improve data accuracy.

### Strengths and limitations

This study has several strengths. It is one of the first to evaluate the accuracy of administrative data (VEMD) against clinician-rated medical records (C-CASA) for suicide attempt classification in EDs. By using a validated classification system (C-CASA) rather than relying solely on ICD clinical coding, the study provides a more reliable benchmark for assessing the VEMD descriptor’s performance. Furthermore, the blinded comparison of C-CASA ratings and VEMD data reduced observer bias, ensuring an objective assessment of agreement and performance. Importantly, the study highlights systemic challenges in suicide attempt detection within ED settings, offering practical insights for improving data surveillance and clinical documentation practices.

Despite these strengths, several limitations must be acknowledged. The study was conducted in a single health network, over a limited time period, during COVID lockdown, which may limit generalisability to other hospitals or populations. Additionally, while C-CASA was applied by a trained clinician, the accuracy of ratings depended on the quality of ED documentation, which is known to be variable and sometimes incomplete (Sveticic et al., 2020). Potential misclassification bias may arise from underreporting in medical records due to clinician workload or patient reluctance to disclose intent. Future research should consider multi-site studies with broader timeframes to assess the generalisability of these findings and explore alternative data-driven approaches for improving suicide attempt detection in emergency settings.

## Conclusion

While the VEMD human intent descriptor reliably confirms suicide attempts, its poor sensitivity limits its usefulness as a screening tool. The significant discrepancies between administrative coding and clinician-rated medical records suggest that suicide attempts are underreported in VEMD, which may impact suicide prevention strategies, health service planning, and data-driven research. Addressing these limitations through improved training, refined coding structures, and advanced data analysis techniques is essential to ensure more accurate and reliable suicide attempt surveillance in ED settings.

## Appendix

**Appendix 1.** C-CASA coding and corresponding VEMD human intent descriptor for diagnostic accuracy comparison.

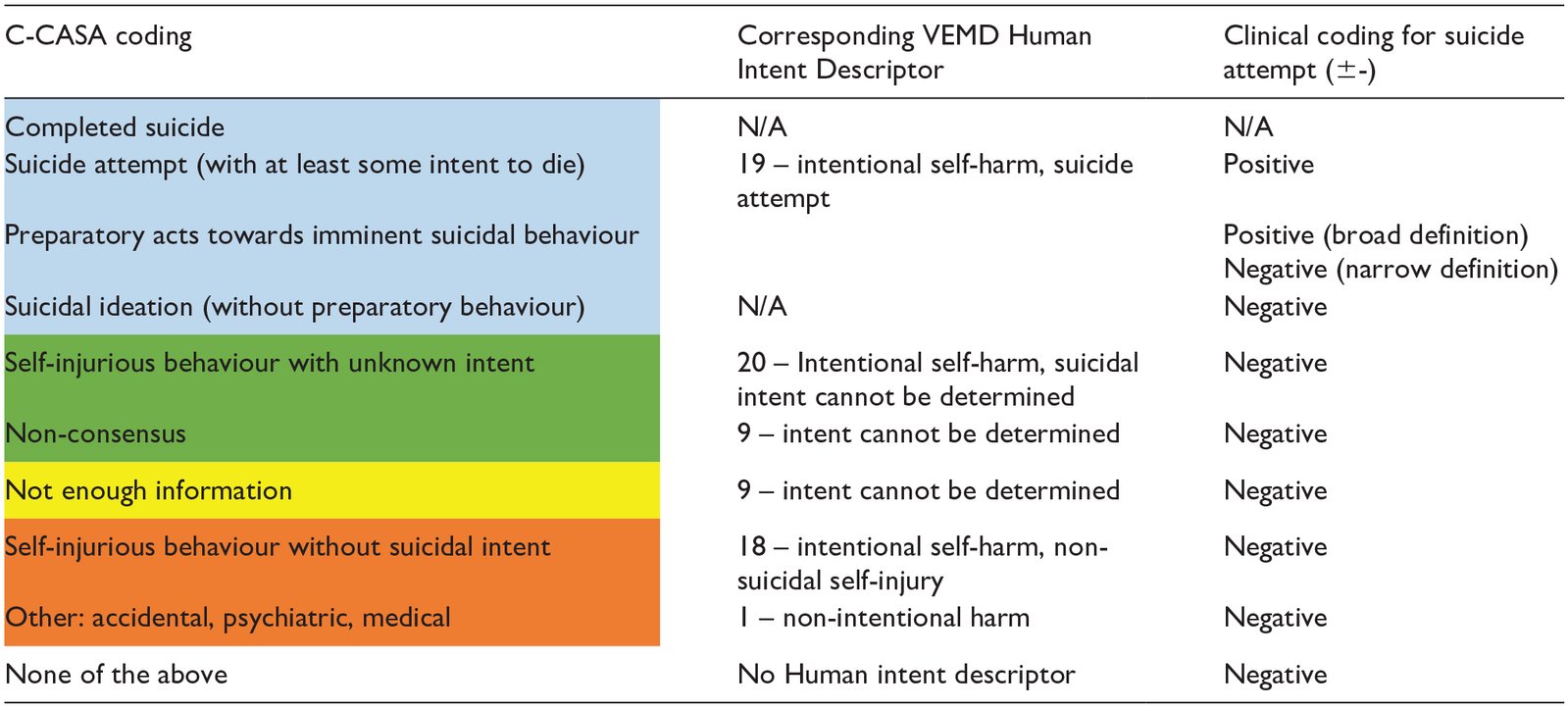

Colour regions reflect the C-CASA suicidality classification scheme as extracted from validation study / systematic review referenced in Figure 1 (Posner et al., 2007).
